# Addressing the post‐COVID era through engineering biology

**DOI:** 10.1049/enb2.12008

**Published:** 2021-06-16

**Authors:** Jennifer Bell, Jim Philp, Richard I. Kitney

**Affiliations:** ^1^ Ekrity Limited Dundalk Ireland; ^2^ Directorate for Science, Technology and Innovation OECD Paris France; ^3^ Department of Bioengineering Imperial College London UK

## Abstract

Currently, the world is faced with two fundamental issues of great importance, namely climate change and the coronavirus pandemic. These are intimately involved with the need to control climate change and the need to switch from high carbon, unsustainable economies to low carbon economies. Inherent in this approach are the concepts of the bioeconomy and the Green Industrial Revolution. The article addresses both issues, but it, principally, focusses on the development of the bioeconomy. It considers how nations are divided in relation to the use of biotechnology and synthetic biology in terms of their bioeconomy strategies. The article addresses, as a central theme, the nature and role of engineering biology in these developments. Engineering biology is addressed in terms of BioDesign, coupled with high levels of automation (including AI and machine learning) to increase reproducibility and reliability to meet industrial standards. This lends itself to distributed manufacturing of products in a range of fields. Engineering biology is a platform technology that can be applied in a range of sectors. The bioeconomy, as an engine for economic growth is addressed—in terms of moving from oil‐based economies to bio‐based economies—using biomass, for example, using selected lignocellulosic waste as a feedstock.

## 1 INTRODUCTION

Many articles are calling for a greener, fairer, more resilient post‐COVID world, from newspapers through several of the most relevant international organisations (e.g. [1, 2, 3]; WEF [4]). To our knowledge, however, the place of biotechnologies and more specifically engineering biology is very often ignored at the political level. The reasons for this are unclear, even though biology and engineering biology may be uniquely flexible for dealing with an ecosystem of grand challenges [5]. Wind and solar technologies target one aspect of future sustainability, and there has been global action to support them in policy. By contrast, engineering biology is a platform technology that addresses many sectors.

What is more, engineering biology can address the ecosystem aspect of grand challenges. For example, future crops will need to feed more people, maintaining high yields while coping with both heat and drought, and often on degraded soils, facing new, unforeseen pests and diseases. Using more mineral fertiliser to maintain yields also means higher emissions and more environmental damage. In other words, addressing one grand challenge will often have negative consequences for others [6]—hence, the ecosystems concept. This interaction is not easily addressed by any technology other than biotechnology.

The apparent ‘biotechnology denial’ is reflected in the existence of at least three bioeconomy types. Vivien et al. [7] discussed three main narratives of the bioeconomy. This article is largely, but not exclusively, focussed on their Type II, a science‐based bioeconomy. In this conception of the bioeconomy, biomass, waste biomass and other sources of carbon, such as waste gases, are used to make new products from materials that might otherwise be lost from the economy. Therefore, this conception is also directly related to their Type III, a biomass‐based bioeconomy. However, our basis for the focus on a science‐based bioeconomy also relates to their Type I, which considers the limits of the biosphere. Thus, we argue that Type II contributes to the other two types: it may also be that this separation to types can be counterproductive at times. This is especially so as the above Type II conception resonates very strongly with the concepts behind the circular economy, that is, of reuse, recycle, repurpose and remanufacture [8]. It should be no surprise that even the way the bioeconomy is defined has diverged over the years (e.g. [9]).

**FIGURE 1:**
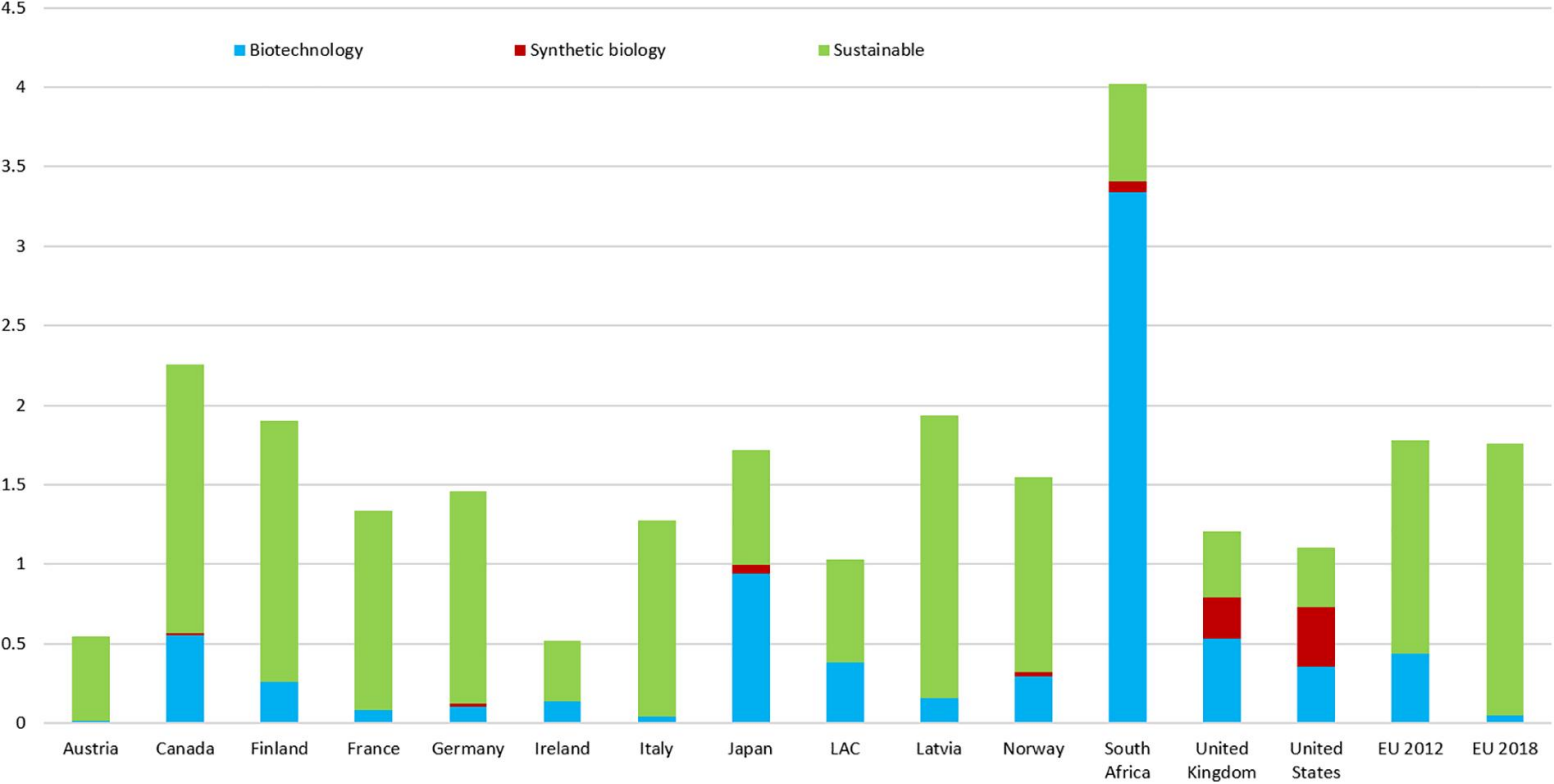
Nations are divided in their use of biotechnology and synthetic biology in their bioeconomy strategies. The figures on the ordinate refer to the number of occurrences of the word per page, excluding footnotes, endnotes and references, of the examined national bioeconomy strategy. LAC, Latin America and Caribbean (from Philip [14])

National bioeconomy strategies or similar instruments have been articulated by at least 50 countries and the European Union. In these, by accident or design there are clearly countries that put biotechnologies, and in some cases engineering or synthetic biology, at the heart of their strategy. This is best seen in the strategies of the United Kingdom and the United States. Specifically, the Royal Academy of Engineering (UK) Synthetic Biology Inquiry Report [10] presented a detailed strategy for the implementation and development of synthetic biology in the UK. This was built upon by two subsequent UK Government Roadmaps, A Synthetic Biology Roadmap for the UK [11] and Bio Design for the BioEconomy [12]. Similarly, in the United States, the Engineering Biology Research Consortium (EBRC) published an extensive, detailed roadmap for the development of synthetic biology/engineering biology—a technical research roadmap [13]. Conversely, there are national bioeconomy strategies where biotechnologies have a much lesser role (Figure 1). Interestingly, between the original bioeconomy strategy of the European Union in 2012 and the update in 2018, the role(s) of biotechnology have been very greatly reduced. Tellingly, the unofficial bioeconomy strategy of Canada [15] proposed to use the bioeconomy definition of the European Union but will ‘rely on biotechnology as a competitive advantage’.

This creates a conundrum for the bioeconomy. As an ‘economy’ of the future, economic sustainability is one of the three pillars of sustainability, along with environmental and social sustainability. There is gathering momentum to the evidence that biotechnology will make huge contributions to the economy in future (see bioeconomy as an engine of economic growth). If so, then the economic windfall may be very unevenly divided, which would contradict the intentions of globalisation (e.g. [16]).

As shown in Figure 1, in terms of national strategies for growing the bioeconomy, these comprise three components biotechnology, synthetic biology (engineering biology) and sustainability. Of the three components, only the United States, the United Kingdom and, to a small extent, Japan, consider synthetic biology to be an important area for the growth of the bioeconomy. In the United Kingdom, for example, Government estimates are that the bioeconomy needs to grow from £220 billion (2016) to £440 billion by 2030 [11]—and synthetic biology/engineering biology is seen as a key driver of this growth. Enabling the advanced bioeconomy through public policy supporting biofoundries in engineering biology is the subject of a recent article [17]. The argument that is made is that there is a real need to address technical challenges in relation to engineering biology in public policy; two important challenges being the need for much higher levels of reproducibility and reliability in terms of biologically based industrial processes, coupled with the need to develop more detailed technical standards. As will be argued later in this article, increasing reproducibility and reliability to industrial standards will require the application of bio design in the context of high levels of automation and automated workflow (e.g. in a biofoundry, usually incorporating AI and machine learning); the application of design optimisation techniques (e.g. Design of Experiments [DoE] and computer modelling). For government funding to respond to the complexity of growing the bioeconomy through engineering biology greater integrated public funding will be required in the areas outlined above. The other important element in the growth of the bioeconomy through engineering biology will be the need to create a specially trained workforce through changes in university and technically‐based education through new programs in community and technology colleges [18].

## 2 THE COMPETITION

We estimate that investment in new biofuels production capacity will take another hit in 2020, well short of the levels implied by existing policy targets, let alone the amounts that would be required to help meet international climate goals [19].

**FIGURE 2:**
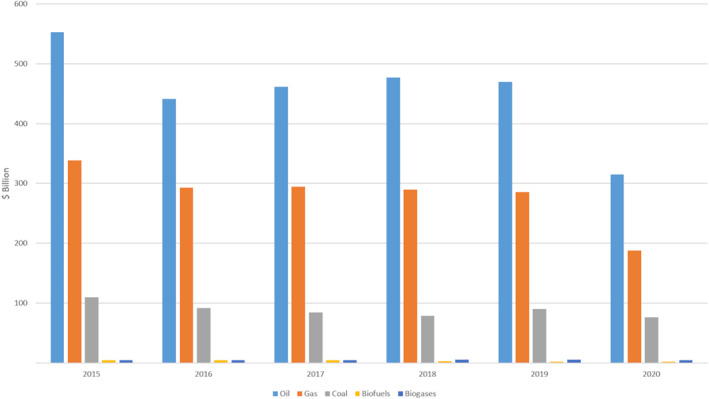
Global energy investments demonstrate the reality of the competition between fossil and renewable energy [19]

Outward appearances indicate a lot of success in climate policy and politics. However, there are fears that post‐COVID emissions will return to previous levels. After a significant drop in global emissions at the start of the pandemic, there was an alarming rebound in the second half of 2020 [20]. Despite all the calls for a greener, more sustainable future, there will be huge temptation for governments to fuel recovery using cheap fossil resources as the world economy infrastructure is fossil‐based [21]. Since the beginning of the COVID‐19 pandemic, governments in G20 countries have committed about a quarter trillion dollars (US) to supporting fossil fuel energy.^1^ International Energy Agency data show the gulf between global fossil and renewable investments (Figure 2).

**FIGURE 3:**
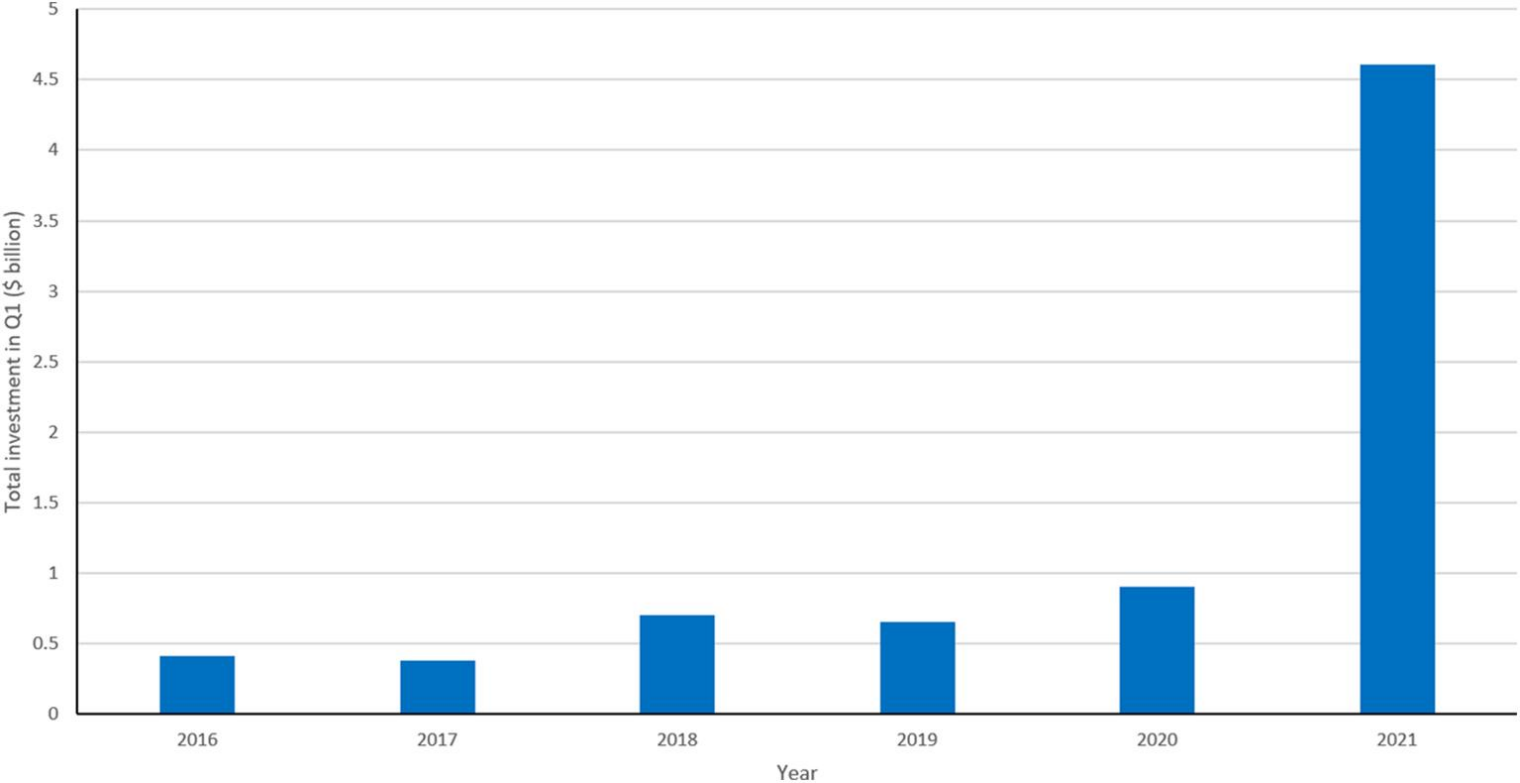
Investments in synthetic biology, by quarter 1 (Q1) results from 2016–2021 (SynBioBETA Market Reports: Redrawn with permission.^2^)

Compare these figures with the investments in synthetic biology (Figure 3). While impressive for a very young discipline with many uncertainties, they are dwarfed by the investments in fossil fuels.

### 2.1 Crude oil's perfect storm

An ‘oil crunch’ has long been debated, when demand for crude oil can no longer be satisfied (e.g. [22]). Several factors betoken a crisis in crude oil supply sooner rather than later. Conventional oil reserves, that is the oil that is plentiful and cheap to extract, have been in decline for decades [23] since at least 1980, but supply has been augmented by unconventional supply, such as shale oil and deep ocean. However, unconventional is a byword for expensive, and deep drilling is considerably more hazardous (e.g. [24]). Low success in exploration and low prices mean that most oil companies are concentrating on improvements in existing fields. Moreover, new finds have been small and small fields deplete at a higher rate than large ones. Add to the list of risks persistent geopolitical risks, especially in the Middle East and Africa, and environmental pressure. The French oil major Total has even committed to becoming carbon neutral in its European businesses by 2050.^3^ Acceptance that fossil fuels are making a large contribution to climate change is at a high. The crunch could make itself evident as early as 2025 [25].

OPEC's spare crude production capacity is likely to narrow by over 60% by 2026, compared to2020,^4^ reducing the ability to ride future shocks to the industry. The most serious and persistent shock could come from the petrochemicals industry as it is predicted to triple in size by 2050 [26], only two innovation cycles from now. While huge oil production losses for fuels due to vehicle electrification creates a crisis for the industry, a tripling of demand from petrochemistry would make up some of the loss. Nevertheless, the current oil industry model is under threat from these seismic changes. During COVID‐19, it has experienced its third price collapse in 12 years [27]. The pandemic has almost monopolised the news in 2020 and 2021; it has almost escaped attention that the oil industry is in its worst crisis of all time [28].

## 3 WHAT ARE THE OTHER SOURCES OF CARBON?

**FIGURE 4:**
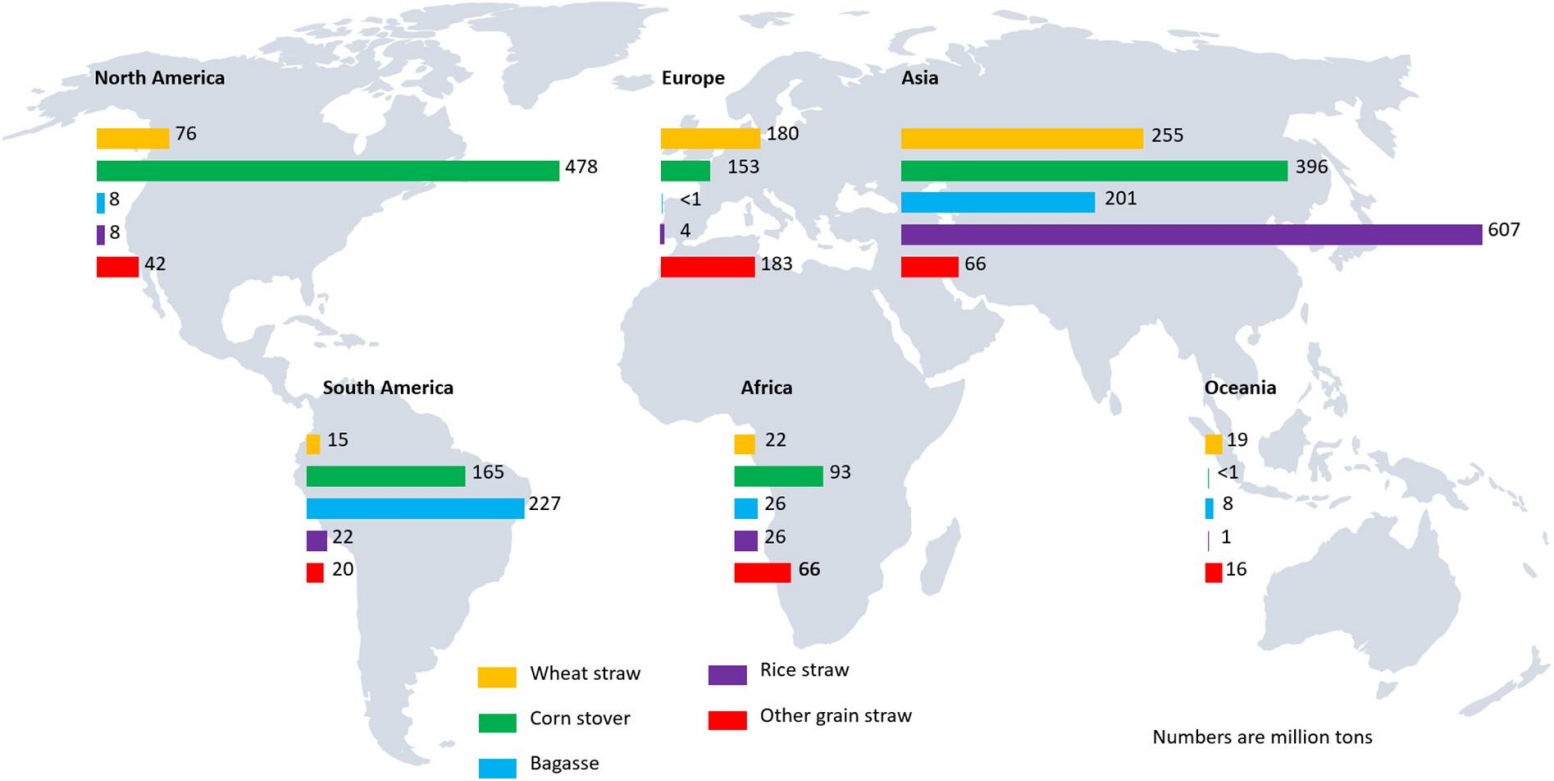
Selected lignocellulosic waste resources [31]

The current consumption of fossil carbon as feedstock for industry production of chemicals, textiles, lubricants and polymers is significantly more than 1 billion tonnes [29]. For the polymers industry alone, the demand for plastics is now of the order of 350 million tonnes.^5^ The only conceivable reserves of carbon large enough to replace fossil carbon are biomass and waste carbon that can be recycled. Typical biomass sources are food and non‐food crops, agricultural residues such as straw, forestry and forestry residues, algae, food waste, wastewater^6^ and specially grown crops such as short‐rotation coppice plantation [30]. Figure 4 shows the theoretical volumes of some of these biomass sources that are available.

In recent years, it has become feasible to utilise waste industrial gases like CO, CO_2_ and H_2_ for fermentation processes. These gases are available in large quantities from a variety of point sources in the industry, for example, cement, steel, chemistry. This leads into the concept of the Green Industrial Revolution and the circular economy.

## 4 BIOECONOMY AS AN ENGINE OF ECONOMIC GROWTH

One example of the importance of the bioeconomy as an engine for economic growth, as described in the introduction of this article, is the UK government's projections for the UK BioEconomy. An important driver will be engineering biology, with a contribution estimated to be around £80 billion [11].

It is well past time for governments around the world to collaborate in developing a standardized and comprehensive understanding of the role of biology in their economies [32].

Various attempts have been made to measure the economic impacts of the bioeconomy, notably in the United Kingdom, the United States and the European Union. These measurements are not easily made, however. The lack of a harmonised definition of the bioeconomy inevitably leads to differences in what is to be measured. The inclusion of human health in the bioeconomy has a large influence, agriculture likewise. Contributions from engineering biology are still small. Nevertheless, it is worth examining some of the published accounts of the size of the bioeconomy to get an overall impression on its importance.

A useful study is that of Carlson [32], who estimated that biotechnology contributes more than 2% of US gross domestic product. In the United States, industries producing bio‐based products (non‐food) represent about 4 million jobs and USD 370 billion [33]. For 2016, data that include indirect and induced multiplier effects suggest the total contribution of the US bioeconomy was almost one trillion dollars [34]. The government of the United Kingdom has projected that the UK bioeconomy needs to grow to £440 billion by 2030 [35]. An important driver will be engineering biology, with a contribution estimated to be around £80 billion, when coupled with more traditional biotechnology.

**FIGURE 5:**
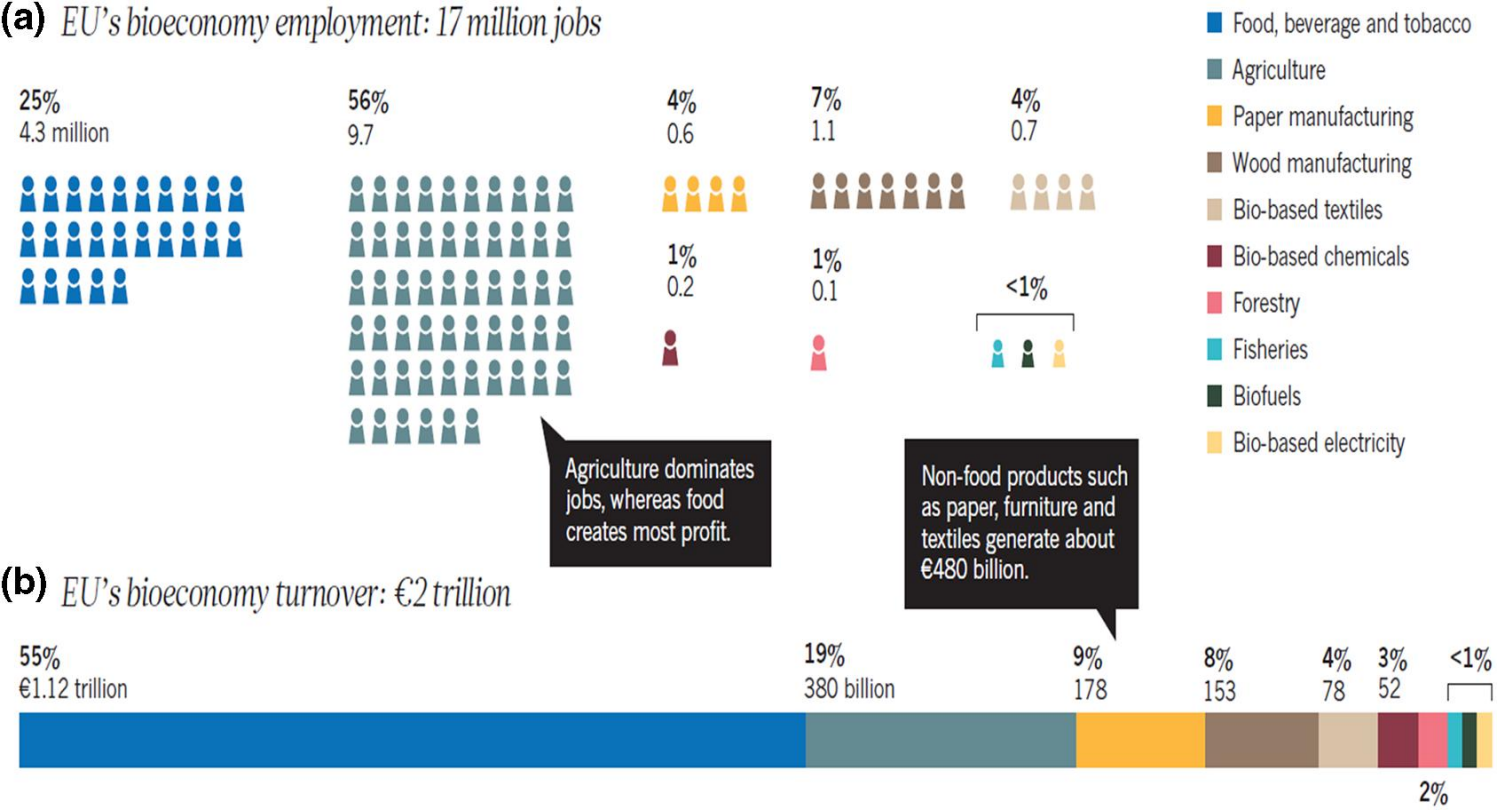
The EU bioeconomy in 2014 (from El‐Chichakli et al. [36])

The breakdown of the contributions to Europe's bioeconomy is insightful. In 2014, the bioeconomy turnover was more than €2 trillion and accounted for 17 million jobs, or 8.5% of the workforce (Figure 5). In Europe, the bioeconomy is still dominated by traditional food and feed products. Non‐food products, such as paper, furniture and textiles account for about €480 billion. The innovative use of biological resources and processes, such as in bio‐based chemicals, pharmaceuticals and plastics, is estimated to have accounted for about €50 billion.

For the future, the study by the McKinsey Global Institute [37] has examined the economic contributions of the ‘bio revolution’ in some detail. It showed the global economic contributions over the near, medium and long term in trillions of US dollars, with that contribution being $4.1 trillion by 2050.

Were the reader to find this section confusing, it is with good reason. The authors have not provided year‐on‐year comparative data for a range of countries because the data are either very difficult to find or they do not exist. What was revealing about the Carlson study was the difficulty encountered in making the calculations. In the United States, the country with the largest biotechnology industry, it was impossible to (economically) distinguish a chemical made through biotechnology and the identical chemical made from fossil resources. This is because there is no North American Industrial Classification System (NAICS) code [38] for these products. The only relevant code is for a subset of pharmaceutical production. Similarly, Ronzon et al. [39] describe estimating ‘bio‐based shares’ for sectors which only partially belong to the bioeconomy, as reported in the European NACE (*Nomenclature Statistique des Activités Économiques dans la Communauté Européenne*) classification.^7^

## 5 KEY ELEMENTS OF THE SYNTHETIC BIOLOGY APPROACH

In terms of having the potential to be a major game changer in relation to the development of bioeconomy, synthetic biology or, as it is often now being called, engineering biology (the two terms are largely interchangeable) has a major potential role to play. A key element in the creation of the field of synthetic biology was the ability to sequence DNA and RNA and, subsequently, to write DNA and RNA chemically (synthesis). These operations effectively became possible about 20 years ago—primarily, but not entirely, because of the human genome project. In the intervening 20 years it has become possible to both read and write DNA/RNA rapidly, reliably and at low cost (relatively low cost in the case of synthesis). These basic operations have opened up the ability to programme cells to produce non‐natural products, for example, to programme yeast cells to produce the direct equivalent to natural spider silk (synthetic spider silk) in industrial quantities. Hence, DNA can be considered as an instruction set that commands the cell to produce a particular product (in the case of the natural DNA, the natural product).

**FIGURE 6:**
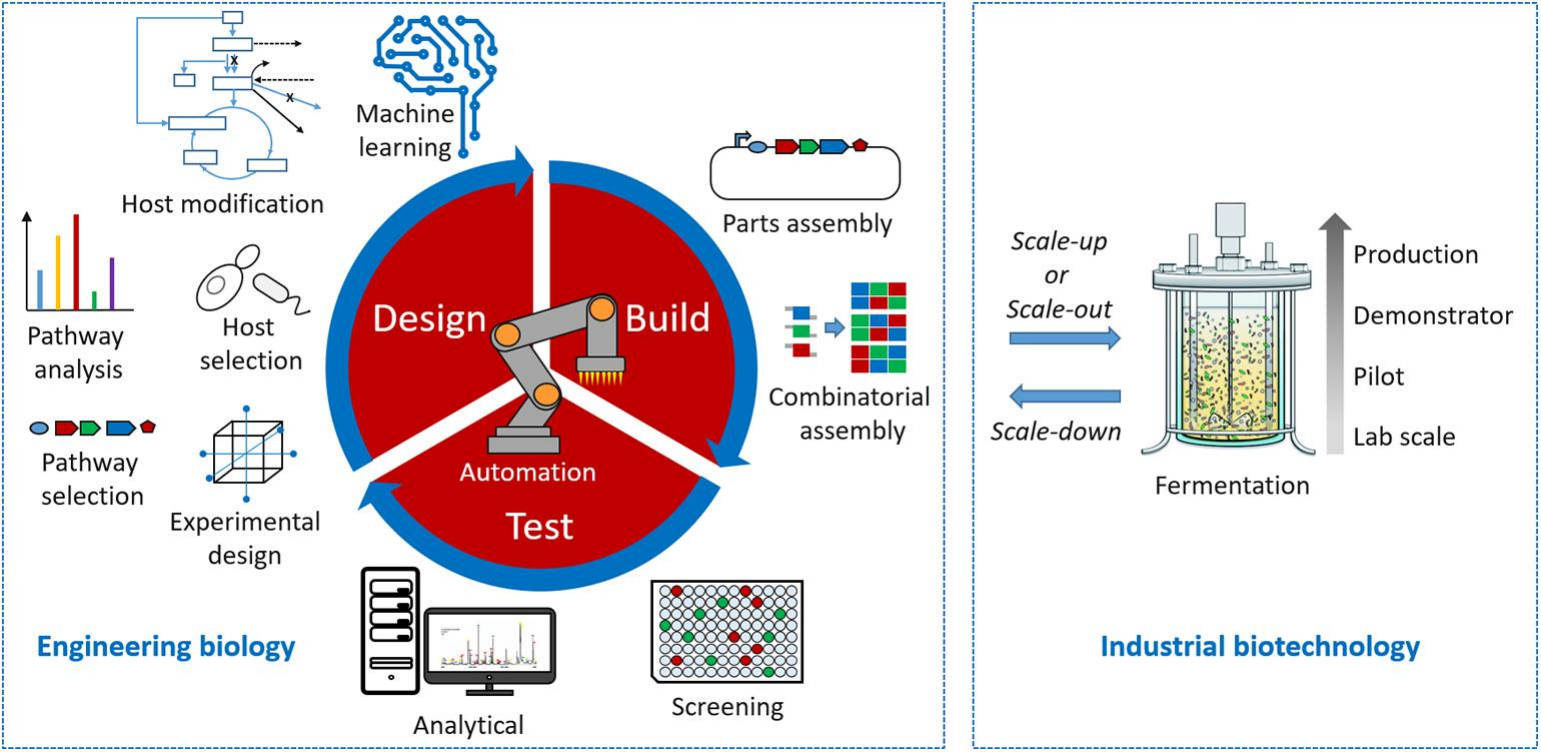
The Biofoundry as the ‘missing link’ in biomanufacturing

On this base has developed the field of synthetic biology/engineering biology that has a number of key elements. In addition to being able to read and write DNA/RNA, at the heart of the field is biodesign and the application of the engineering principles of characterisation, standardisation and modularisation. This is coupled with the synthetic biology design cycle that comprises specifications, design, modelling (dry lab), and implementation, testing and validation (wet lab) and debugging (wet/dry lab). This is encapsulated in the paradigm design, build, test and learn (or DBTL) (Figure 6). This whole approach is the basis of digital biology (normally referred to as synthetic biology or engineering biology).

Today, the two key differentiators of the approach are the application of bio CAD design tools and the implementation of automation, AI and machine learning (currently, usually in the form of a biofoundry). Another basis for synthetic biology/engineering biology is the implementation of an alternative industrial model. The traditional industrial model comprises oil‐based feedstocks as its input, feeding through synthetic chemistry to industrial processes and products. The alternative industrial model, based in synthetic biology/engineering biology, comprises bio‐based feedstocks feeding through synthetic biology/engineering biology to industrial processes and products [40].

Digital biodesign, often using CAD tools, and automation, AI and machine learning implemented in biofoundries are a major game changer. In terms of implementation, engineering biology seeks to increase reproducibility to enable the quantitative precision required for modern manufacturing. Standards, automation, and machine learning are key to the success of this approach. It is an approach applicable to both research and industrial production. Scale‐down refers to acquiring data at the production scale and transferring the information back to the laboratory via scale‐down simulators. If need be, the production strain can be re‐engineered and/or new information is back‐translated to the fermentation operation for its fine‐tuning.

The approach, however, is inhibited by a lack of rigorous modelling and computation. Additionally, the transition to multi‐thousand litre bioreactor processes alters the conditions greatly from those of the laboratory, for example, oxygen concentration gradients, changes in pH, shear forces on cells, and even impurities in lower‐cost culture media. Industrial scale production has its own specific and potentially expensive requirements that can be addressed by biofoundry operations at a distance from a production site. A feature of the biofoundry approach consistent with modern manufacturing is that the site of the design (the biofoundry) can be totally separated from the site of manufacturing (typically the biorefinery).

Synthetic biology/engineering biology is a platform technology that can be applied across a wide range of fields. However, the basic DBTL/design cycle approach lends itself readily to distributed design and manufacture. The power of the engineering biology/digital biology approach is that each stage of the design cycle can be decoupled and distributed to centres around the world. Similarly, once the design has been completed manufacturing can be undertaken at different highly automated manufacturing facilities that may well be widely geographically distributed.

**FIGURE 7:**
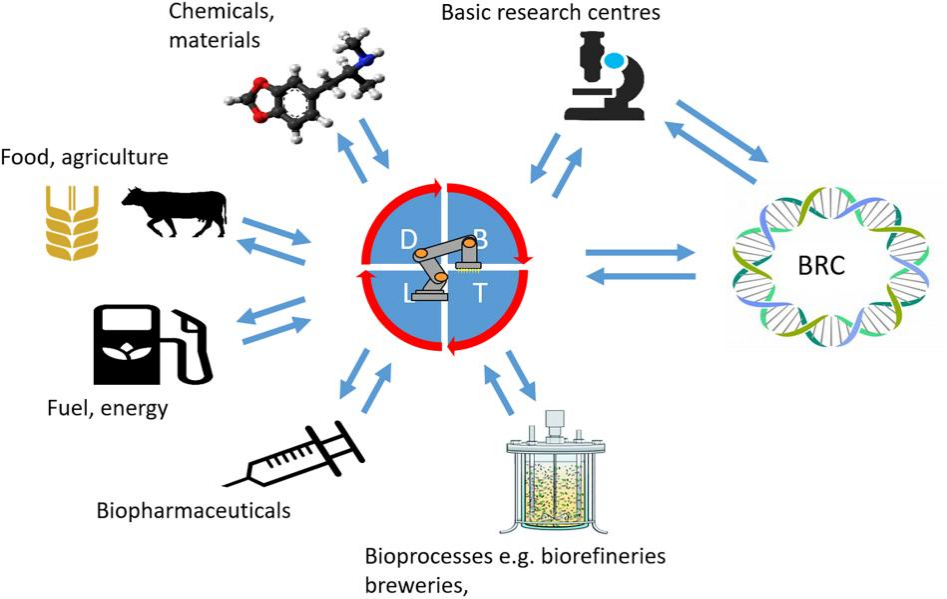
Engineering biology platforms address a wide range of sectors. BRC, biological resource centre; DBTL, design–build–test–‐learn cycle (characteristic of biofoundries)

A particularly powerful combination could be the interaction between biofoundries and the modern biological resource centres (BRCs), which can be described as the curators of the known biology. In other words, this is the combination of the known biology (from sequencing) with the new biology (from biofoundries) (Figure 7). Sequencing initiatives such as the Earth Biogenome Project,^8^ which aims to sequence the genomes of all known eukaryotic organisms [41], will mean a large increase in the availability of genome sequence for use in products over a wide range of sectors.

### 5.1 Problems with the current manufacturing methods

Fundamentally, many of the problems associated with biomanufacturing processes arise from the fact that they are largely an analogue process. This means that, for example, uniformity of design manufacture is difficult to achieve. Conversion of the design and manufacturing process to the digital domain through the combination of the engineering biology approach, coupled with high levels of automation, not only leads to a much more targeted exploration of the design space but also increases reliability and reproducibility.

The ability to design, build, test and learn largely in the digital domain—with implementation in the wet lab or, increasingly, hybrid implementation in the biofoundry (a combination of wet and dry operation) leads directly into the concept of the design of biological products at one or more locations, coupled with distributed manufacturing. A recent example of this approach is the development of mRNA vaccines. These are much more amenable to both distributed manufacture and rapid modification using engineering biology principles and techniques. It is, therefore, feasible, in terms of this and other examples, to develop a design in one or more locations and to then distribute it digitally to manufacturing units that are widely geographically dispersed. In the case of RNA vaccines, the number of doses that can be derived from a litre of the vaccine “broth” far exceeds that of DNA‐based vaccines. Consequently, distributed manufacture becomes much more feasible.

## 6 PHARMACEUTICAL INDUSTRY RECOVERY FROM COVID‐19

Like many industries, the pharmaceutical industry is examining its recovery from the COVID‐19 pandemic. This pandemic has accelerated the industry's and regulator's decision‐making. Solutions relate to all pharmaceutical companies, that is, bio‐based and non‐bio based.

McKinsey & Company lists pharmaceutical industry considerations under companies, industry and governments [42]. Consideration focus areas include supply chain and asset reorganisation, agility, transparency, new technologies and shaping a workforce and capabilities to deal with more remote and distributed tasks.

The pandemic has particularly challenged the business continuity frameworks of all companies. Recipharm’s executive vice president of corporate development, Mark Quick, explained that early in the pandemic there was a shortage of active pharmaceutical ingredients caused by Chinese factory closures [43]. Recipharm is a contract drug development and manufacturing organisation. Mark Quick said that because of Chinese company closures, ‘many governments are now encouraging their local pharma sector to localize supply chains—or at least diversify them—to safeguard against disruption during future economic shutdowns’ [43].

### 6.1 Local essential medicines production

Throughout the pandemic a light has shone on the uneven global distribution of manufacturing technology and skills. News reports discuss how the global population needs to be vaccinated to eradicate risks of more waves of infection. Medically underserved communities are on a platform that everybody can see. The pandemic has also highlighted vaccine supply tension in countries that are not considered medically underserved.

The United Nations Conference on Trade and Development (UNCTAD) began drafting an international code on the transfer of technology in the 1970s [44]. Technology transfer was defined as ‘transfer of systematic knowledge for the manufacture of a product, for the application of a process or for the rendering of a service and does not extend to transactions involving the mere sale or mere lease of goods’ [44].

In 2011, the World Health Organization published a report called ‘Local Production for Access to Medical Products: Developing a Framework to Improve Public Health’. The report was an output from a project called ‘Improving access to medicines in developing countries through technology transfer and local production’. The Department of Public Health Innovation and Intellectual Property of the World Health Organization (World Health Organization/PHI) implemented the project. They did this in partnership with the United Nations Conference on Trade and Development and the International Centre for Trade and Sustainable Development (ICTSD), with funding from the European Union. In fact, local production was discussed in 1978 at the International Conference on Primary Health Care [45, 46].

Sub‐Saharan African governments view local pharmaceutical production as a way to promote technology transfer, building capacity and improving access to essential medicines [46]. For example, Nigeria's National Agency for Food and Drug Administration and Control gives a newly registered imported product a maximum of 10 years to migrate to local production as of May 01, 2019—failing to do so cancels the product's registration [47]. Local medicine production will seed business ecosystems in communities.

## 7 FROM THEN TO NOW

For thousands of years humans intervened to selectively breed animals and plants for agriculture to feed their communities. Engineering biology allows human intervention at more immediate speeds compared to selective breeding, although decisions to go ahead with it are complex [48].

Drought‐resistant genetically modified crops are grown in the United States. With regulatory consideration salt, heat and drought, low nutrient, and pest‐tolerant genetically modified crops could be grown to ensure global food security [49]. Engineering biology allows meat to be produced in vitro [50]—meat without slaughter. There is a perception that genetic modifications are a threat; equally, genetic modifications are already being used [48]. The pharmaceutical industry produces medical treatments using transgenic animals [51, 52]. The first veterinary DNA vaccine was authorised in Canada in 2005 [53].

Biomedical science advances include genetic cell‐based and tissue‐engineered products (TEPs) under advanced therapeutic medicinal products (ATMPs) [54]. In the United States, these products are categorised as cell and gene therapies (CGTs).

Commercial cell and gene therapy developers contributed to a survey [55]. Most of the developers were small‐ to medium‐sized enterprises. A literature review and survey highlighted the challenges related to the following:

‐ Addressing regulatory requirements
‐ Complex manufacturing processes
‐ Funding difficulties
‐ Heterogeneous national procedures at member state level
‐ Implementation of Good Manufacturing Practices (GMP)
‐ Intellectual property
‐ Skilled resources and knowledge gaps
‐ Manufacturing standardisation
‐ Public perception

A January 2021 press release informed how German researchers used gene therapy to stimulate nerve cells to produce a protein to cure mice of paraplegia in two to three weeks [56]. Macquarie University Department of Biomedical Sciences researchers [57] found a “Trojan horse” strategy that helps RNA interference therapy in neurodegenerative diseases like Alzheimer's disease.

## 8 BIOMEDICAL SCIENCE IS NOT CLEARLY PART OF THE GLOBAL BIOECONOMY

Biomedical science is outside the remit of the engineering biology economic sector in Europe even though biomedical science and engineering biology share many of the same bio‐based technologies. There is regulatory cross‐over in Europe as ATMP authorisations fulfil genetically modified organism (GMO) regulatory requirements [58]. The EU GMO legislation was written 20 years ago mainly to regulate agricultural activity to protect food consumers and the environment [58, 59].

There are difficulties in receiving authorisation for ATMPs considered to contain GMOs [58, 59, 60]. The Court of Justice of the European Union decided that organisms obtained by directed mutagenesis are considered GMOs in July 2018 [61, 62]. As a result, their producers are obliged to fulfil the requirements in the European GMO Directive.

Shortly after the Court of Justice of the European Union decision, the European Commission Scientific Advice Mechanism Group of Chief Scientific Advisors released a statement saying that the GMO Directive should be revised ‘to reflect current knowledge and scientific evidence, in particular on gene editing and established techniques of genetic modification’ [63]. Legislation in the USA considers non‐binding recommendations where GMO environmental risk assessment is not required for medicines such as gene therapies, vectored vaccines, and related recombinant viral and microbial products [64].

Clinical trial authorisation for ATMPs, including vaccines and other COVID‐19 treatments, has additional steps to comply with the GMO legislation [58]. The EU made a decision to temporarily exempt potential vaccines and treatments from some GMO legislated requirements in recognition of complications leading to delays of clinical development [58]. A European Commission press release (2010) stated, ‘Time is of the essence. Every month gained … saves lives, livelihoods…’. This statement could be applied to a broader range of medicinal products [65].

## 9 ENGINEERING BIOLOGY IS HIGHLY DISRUPTIVE TECHNOLOGY

An alternative model using bio‐based feedstocks and synthetic biology leads to industrial processes and products. This means that, potentially, local bio‐based feedstocks can be used as the input to a manufacturing process.

The drive to a low carbon economy is likely to have significant implications in relation to traditional manufacturing processes and supply chains not just in the healthcare sector but across other sectors of the economy. ‘Disruptive’ can be interpreted in several ways, and this section will examine these by examples.

### 9.1 Drop‐in replacement products

The (eventual) replacement of the oil barrel represents one of the largest challenges of the times and is likely to result in new industries and technologies, with incumbent fossil energy companies necessitated to change their business models radically or to disappear. The chemical industry currently makes around 70,000 products.

Adipic acid is a classic example. It is one of the most important small molecules in the modern chemicals industry, an intermediate in the production of nylon. Industrial production of adipic acid relies on fossil feedstocks and produces large amounts of nitrous oxide, a greenhouse gas (GHG) three hundred times more potent than carbon dioxide. Suitor et al. [66] described the first synthesis of adipic acid from guaiacol, a lignin‐derived feedstock, in the biotechnology industry workhorse bacterium *Escherichia coli*. Effectively, lignin is a waste, and its conversion to adipic acid using synthetic biology keeps it in circulation, thereby contributing both to the bioeconomy and the circular economy.

### 9.2 Totally new products and materials

True disruption occurs with a totally new product or service displacing an earlier one.^9^ Spider silk is an example of a material that could be disruptive in many applications. Stronger than steel, tougher than Kevlar but also flexible, the range of applications is large. They are lightweight and virtually invisible to the human immune system, giving them “revolutionary potential” for medicine and the industry [67]. Among the newer applications of spider silk being considered are microphones in hearing aids and cell phones. The German company AMSilk has entered into an agreement with Airbus to develop structural materials for aircraft using synthetic spider silk. A biodegradable shoe has been developed by Adidas using this material. Silk has high‐value applications in cosmetics, and Givaudan has acquired the cosmetics business of AMSilk.

Engineering biologists are interested in spider silk since there are many gene and protein candidates for transgenic studies. This implies the possibility of tailor‐making different spider silks for different materials and applications [68]. However, working with spiders as factories is impracticable, and the goal is to make spider silks in microorganisms. There are many technical barriers, but this makes the task one that is really suitable for the engineering biology approach [69]. Moreover, these materials are dislocated from mining industries.

### 9.3 Changing how industries work

#### 9.3.1 Distributed manufacturing

Centralisation of labour and production has been the norm in many industries, but in 2015 the World Economic Forum put distributed manufacturing in its top 10 emerging technologies for the year.^10^ In essence, production is done close to the final customer and much of the material supply chain is replaced by information [70]. It looks unsuited to high production volume industries, such as commodity chemicals and automotive, but is more feasible for low‐volume, higher added value products and value chains [71]. However, the biofoundry concept is suited to this model. The design can be done from any location, even from home. The design can be transferred to the biofoundry electronically, and the prototyping is done in the biofoundry to optimise the design and make prototypes. Then the information and prototypes can be transferred to a production facility, and subsequently, electronic exchanges continue. The vaccines' example illustrates the possibilities well.

#### 9.3.2 The mobilisation of wastes to secondary raw materials

However, new conversion processes are needed to improve chemical and thermal properties and increase the energy densities of these feedstocks if they are to replace those derived from petroleum [72].

Much of the future development of the bioeconomy is predicated upon the processing of biomass resources into bio‐based energy, fuels, chemicals and materials. However, a large component of this feedstock is lignocellulose, which is recalcitrant to bioprocessing [73] and thus, currently much of the pre‐treatment of biomass for a biorefinery relies on chemical and/or physical/thermal treatments.

**FIGURE 8:**
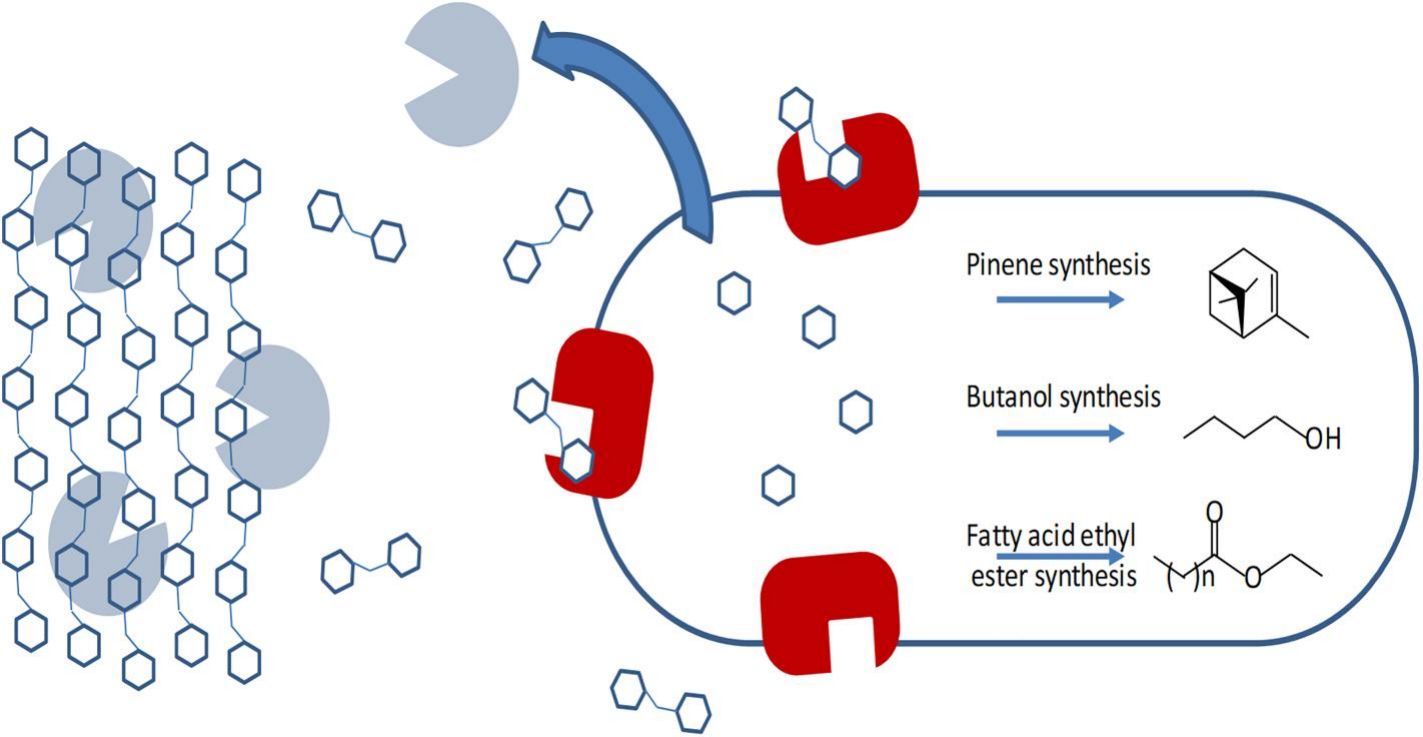
Engineering *E. coli* for use in CBP. Cellulose and hemicellulose were hydrolysed by secreted cellulase and hemicellulose enzymes into soluble oligosaccharides (blue). *β*‐glucosidase enzymes (red) further hydrolysed the oligosaccharides into monosaccharides, which were metabolised into three different biofuels (Bokinsky et al. [76]: reproduced with the permission of the authors)

Consolidated bioprocessing (CBP) is a system in which enzyme production, substrate hydrolysis, and fermentation are accomplished in a single process step by lignocellulolytic microorganisms [74]. An obvious advantage is that it reduces the number of unit processes in an overall bioprocess [75]–the more work that can be done by the microbial catalyst, the less that has to be done with mechanical and chemical engineering. An example by Bokinsky et al. [76] demonstrates the potential (Figure 8).

Of the various possible bioprocessing technologies, CBP may be the most economical in the long run, but productivity is still lacking [77]. This might present an opportunity for the engineering biology approach. Indeed the complexity of the tasks to engineer all the different functions into a single microbial biocatalyst could have been tailor‐made for the iteration of the DBTL cycle in the biofoundry [78].

For many decades waste industrial gases have been a point and dispersed pollution problem of large scale in industries such as cement and steelmaking. The microbial fermentation of waste industrial gases such as CO, CO_2_ and H_2_ to useful products such as ethanol [79], bioplastics [80] and animal feed [81] is becoming a reality. Synthetic biology and metabolic engineering approaches will play essential roles in expanding the product spectrum beyond ethanol to other fuels and commodity chemicals [82]. The microbes concerned need significant genetic engineering to make a bioprocess viable, and the engineering biology approach is most suited to the rational design of such microbes.

#### 9.3.3 Genomics has revolutionised dairy farming

Genomic evaluation is the process of producing estimates of genetic merit based on the DNA information of an animal, depending on what traits are of interest, for example, fat content and protein content [83]. This is not about genetic modification but accumulating genomic data that relate to traits. Genomic selection allows breeders to identify genetically superior animals at a much earlier age [84], thereby increasing the cost‐efficiency of traditional breeding programmes. A study by Garcia‐Ruiz et al. [85] definitively showed that the rate of gain in significant traits has accelerated when comparing selective breeding programmes before and after genomic selection was introduced.

#### 9.3.4 Aquaculture replaced antibiotics with vaccines

Fish farming now produces around 50% of the fish consumed globally and has made a major contribution to food security. In the early history of the farmed salmon industry, now Norway's second‐largest sector, antibiotics were used very widely to control diseases. However, excessive use of antibiotics is thought to contribute to the spread of drug‐resistant pathogens in both farmed animals and wild fish, with a potential risk for humans [86].

A biotechnology alternative to antibiotics is vaccination. By the 1990s, the Norwegian salmon industry had all but consigned the antibiotic era to history.^11^ Fish vaccines have been produced by rather conventional vaccine technology in the past. Like with human vaccines [87], biotechnology and synthetic biology approaches to new fish vaccines show the greatest potential for further improvements [88].

## 10 HOW TO FUND THE REVOLUTION

The bio‐based revolution is a systemic change akin to the transformation from wood to coal and then coal to oil [29]. But climate change is forcing the speeding up of the transformation. Naturally, this is a vast global effort of immense financial cost. However, there are mechanisms to fund this transformation directly related to climate change mitigation.

### 10.1 Explicit carbon price and carbon tax

Economic penalties in the form of a tax on carbon emissions appear as an obvious policy measure. As of 2019, there were 57 carbon prices either in practice or in development. This represents some 11 gigatons of CO_2_ equivalent, or 20% of global emissions per annum, and the figure is steadily rising [89]. However, the level of tax is nowhere near high enough: the average price of emissions worldwide is only 2 USD per tonne, when a realistic level would be around 70 USD per tonne [90].

There are essentially two methods for using revenues from these taxes to help grow the bioeconomy. In the first, revenues are added to the general budget of a government and that government can choose to use these revenues for climate‐friendly purposes. Alternatively, the revenues can be earmarked for specific projects or purposes, rather than being added to the general budget. Both approaches have advantages: adding to the general budget minimises the cost of new administration, while earmarking is more direct, transparent, and perhaps easier for gaining public acceptance.

### 10.2 Radical reform of fossil fuel subsidies

**FIGURE 9:**
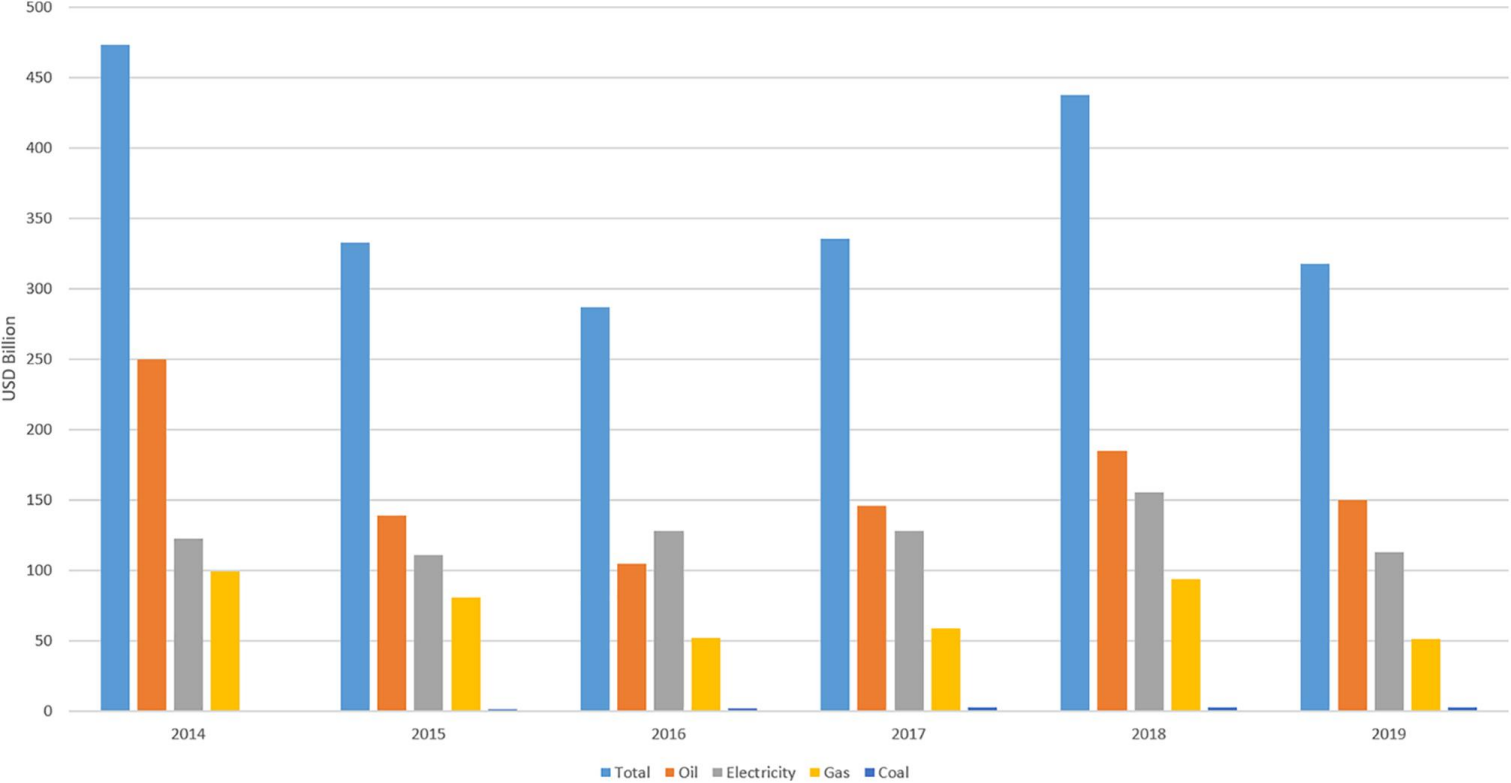
Global fossil subsidies from 2014–2019 (from IEA [91])

Subsidies for emerging industries are supposed to end at some stage when the industry is considered to be self‐supporting. And yet, fossil fuel subsidies represent perhaps the largest subsidy system of all time (Figure 9)—and the industry is over a century old. Most of these subsidies are inefficient and wasteful, but political backlash against their removal makes reform difficult [92]. Reform of these subsidies could see them used to help fund renewable technologies such as engineering biology.

## 11 SUMMARY AND CONCLUSIONS

The article covers a range of issues related to addressing the post‐COVID era. The basis of the paper is that the world must address two fundamental issues of major importance—climate change and the coronavirus pandemic (and the possibility of further pandemics). We make the argument that fundamental to the first of these issues, namely climate change, there is a pressing need to move from high carbon, unsustainable economies to low carbon economies.

A key driver of this change will be the development and application of technology based on synthetic biology/engineering biology. The field has developed over the last 20 years, based on the ability to read and write (chemically) DNA and RNA. This has resulted in the ability to undertake bio design and its implementation in facilities with high levels of automation (including AI and machine learning)—fundamentally, the world of digital biology. The use of these techniques is fundamental to a new industrial model comprising bio‐based feedstocks as its input, feeding through synthetic biology/engineering biology to industrial processes and products.

The techniques and applications of high levels of automation mean that in bio manufacturing much higher levels of reliability and reproducibility can be achieved. In addition, this also lends itself to distributed manufacturing across a range of fields. One example is the ability to design vaccines (particularly mRNA vaccines) at one or more locations and to then distribute their manufacture across the world at numerous sites in many countries—through the application of synthetic biology/engineering biology techniques (digital biology).

Our conclusion is that there is now the potential to enter a new world of low carbon economies, coupled with systematic bio design and bio manufacturing—through synthetic biology engineering biology—that lends itself to distributed manufacturing [87].

## CONFLICT OF INTEREST

No conflicts of interest.

## Footnotes

^1^

https://www.energypolicytracker.org/region/g20/

^2^
Synbiobeta Market Reports, 7 April 2021. https://synbiobeta.com/wp‐content/uploads/2021/04/SynBioBeta‐Market‐Report‐Q1‐2021.pdf?vgo_ee=VIxEklLTzVTZ2S9GfDCfQHMCbfnOZGgcei5S2nhkvNY%3D

^3^

https://www.total.com/media/news/total‐adopts‐new‐climate‐ambition‐get‐net‐zero‐2050

^4^

https://www.spglobal.com/platts/en/market‐insights/latest‐news/oil/031721‐rising‐oil‐demand‐to‐outpace‐opec‐supply‐growth‐shrinking‐spare‐capacity‐cushion‐iea

^5^

https://ourworldindata.org/plastic‐pollution

^6^

https://www.energy.gov/eere/bioenergy/biomass‐resources

^7^

https://ec.europa.eu/eurostat/statistics‐explained/index.php/Glossary:Statistical_classification_of_economic_activities_in_the_European_Community_(NACE)

^8^

https://www.earthbiogenome.org/

^9^

https://en.wikipedia.org/wiki/Disruptive_innovation

^10^

https://www.weforum.org/agenda/2015/03/top‐10‐emerging‐technologies‐of‐2015‐2/

^11^

https://www.who.int/features/2015/antibiotics‐norway/en/
